# Editorial: Embodying the Self: Neurophysiological Perspectives on the Psychopathology of Anomalous Bodily Experiences

**DOI:** 10.3389/fnhum.2017.00631

**Published:** 2017-12-19

**Authors:** Mariateresa Sestito, Andrea Raballo, Giovanni Stanghellini, Vittorio Gallese

**Affiliations:** ^1^Department of Psychology, Wright State University, Dayton, OH, United States; ^2^Unit of Physiology, Department of Neuroscience, University of Parma, Parma, Italy; ^3^Department of Psychology, Psychopathology and Development Research, Norwegian University of Science and Technology (NTNU), Trondheim, Norway; ^4^DiSPUTer, “G. d'Annunzio” University of Chieti-Pescara, Chieti, Italy; ^5^Department of Cognitive Science and Language, Diego Portales University, Santiago, Chile; ^6^Institute of Philosophy, School of Advanced Study, University of London, London, United Kingdom

**Keywords:** anomalous experiences, body-object, body-subject, embodiment, mirror neurons system, neurophysiology, phenomenology, psychopathology

Since the beginning of the twentieth Century, phenomenology has developed a distinction between lived body (*leib*) and physical body (*koerper*), a distinction well known as body-subject vs. body-object (Hanna and Thompson, 2003). The lived body is the body experienced from within—my own direct experience of my body lived in the first-person perspective, myself as a spatiotemporal embodied agent in the world. The physical body on the other hand, is the body thematically investigated from a third person perspective by natural sciences such as anatomy and physiology.

An active topic affecting the understanding of several psychopathological disorders is the relatively unknown dynamic existing between aspects related to the body-object (that comprises the neurobiological substrate of the disease) and the body-subject (the experiences reported by patients) (Nelson and Sass, 2017). A clue testifying the need to better explore this dynamic in the psychopathological context is the marked gap that still exists between patients' clinical reports (generally entailing disturbing experiences) and etiopathogenetic theories and therapeutic practices, that are mainly postulated at a bodily/brain level of description and analysis. The phenomenological exploration typically targets descriptions of persons' lived experience. For instance, patients suffering from schizophrenia may describe their thoughts as alien (“thoughts are intruding into my head”) and the world surrounding them as fragmented (“the world is a series of snapshots”) (Stanghellini et al., 2015a). The result is a rich and detailed collection of the patients' qualitative self-descriptions (Stanghellini and Rossi, 2014), that reveal fundamental changes in the structure of experiencing and can be captured by using specific assessment tools (Parnas et al., 2005; Stanghellini et al., 2014; Sass et al., 2017).

The practice of considering the objective and the subjective levels of analysis as separated in the research studies design has many unintended consequences. Primarily, it has the effect of limiting actionable neuroscientific progress within clinical practice. This holds true both in terms of availability of evidence-based treatments for the disorders, as well as for early diagnosis purposes. In response to this need, this collection of articles aims to promote an interdisciplinary endeavor to better connect the bodily, objective level of analysis with its experiential corollary. This is accomplished by focusing on the convergence between (neuro) physiological evidence and the phenomenological manifestations of anomalous bodily experiences present in different disorders. Still indeed, little effort has been channeled in order to plan comprehensive research protocols that include aspects derived from the lived world of patients.

The idea of addressing the human body going beyond the simple Hippocratic idea is revitalized in the concept of *Embodied Medicine* proposed by Riva et al. Body representation is a complex aspect, as it involves the encoding and integration of a wide range of multisensory—somatosensory, visual, auditory, vestibular, visceral—and motor signals (Blanke, 2012). Specifically for self-bodily recognition, behavioral and anatomical data suggest that implicit and explicit routes for self-body knowledge are dissociated and mediated by different cerebral networks at a brain level (Candini et al.). The concept of *Embodied Medicine* takes advantage from the multisensory nature of the body and promote the use of advanced technologies for altering the experience of being in a body, with the goal of improving health and well-being. In particular, the technology proposed by Riva et al. works as a means to modify the inner body for treating different neurological and psychiatric diseases and their phenotypical manifestations. The commentary of Pistoia et al. in this respect, illustrates other potential applications of this technology specifically in the context of neurological disorders like the Locked-in Syndrome.

The contribution from Northoff and Stanghellini outlines an experimentally testable hypothesis meant to provide converging evidence from psychopathological facts (phenomenology, see Stanghellini et al., 2014) and neurophysiological measures in schizophrenia. This is accomplished by combining temporal measures of the brain's spontaneous activity of interoceptive stimuli and temporal measures for the subjective experience of the body. Along similar lines, the work of Ebisch et al. supports the existence of a brain network processing the integration of information derived from multiple sources during social perception. Authors here hypothesize that such integrative processing of social information occurring at a brain level might be mediated by the linking of external stimuli with self-experience.

An empirical attempt to find a common structure that integrates intero- and exteroceptive stimuli processing can be found in other articles included in this collection. In their study, Ardizzi et al. consider the individual sensitivity to detect the visceral sensations originating inside of the body (i.e., interoception accuracy) as a facet of self-integrity in schizophrenia. The results report a reduced sensitivity in patients to their inner bodily signals, that correlates with positive symptoms severity.

Numerous studies show that interoception is altered in different psychiatric disorders. For example, low interoceptive accuracy was established in anorexia nervosa (Pollatos et al., 2008; Stanghellini et al., 2012, 2015b; Gaudio et al., 2014), major depression (Furman et al., 2013; Harshaw, 2015) and depersonalization- derealization disorders (Sedeño et al., 2014). Ambrosecchia et al. report how interoception and autonomic regulation are modulated during social interactions in a population of restrictive anorexia nervosa patients. Authors suggest that an autonomic imbalance and its altered relationship with interoception might have a key role in emotional and social disposition manifestations of the disorder. In their article, Pollatos et al. report how anorexia patients show a significant decrease of interoceptive accuracy during self-focus sessions, a therapeutic practice aimed at increasing attention to patients' own bodily features. This study provides insights into phenomenological aspects related to body-avoidance feelings that characterize anorexia, and draws attention to possible implications for treatment.

Anomalous bodily experiences may also accompany a number of chronic pain conditions. The work from Tajadura-Jiménez et al. describes how acoustic sensory feedback can alter humans' body perception and the pain experienced, suggesting potential practical applications in the clinical setting. In their study, Valenzuela-Moguillansky et al. highlight possible interactions between exteroceptive and interoceptive self-body awareness aspects in patients suffering from fibromyalgia. Authors then relate these aspects to pain, suggesting suitable therapeutic practices tapping into this interaction.

Specifically in the context of schizophrenia, however, genetics still remain a crucial risk factor. The work of Henriksen et al. reviews the state of the art of the complex genetic architecture of schizophrenia and related phenotypes evident in clinical practice. Empirical research on anomalous self-experiences reported by patients with schizophrenia (Parnas and Handest, 2003) indeed, considers this aspect to be an intermediate phenotype of the disorder. Investigating the neurophysiological correlates of anomalous self-experience became a topic of intense research. Some studies for example, point toward a disturbance of emotional motor resonance and multisensory integration as body-level correlates of anomalous self-experience in schizophrenia (Sestito et al., 2013, 2015a,b; Gallese and Ferri, 2014; Ebisch and Gallese, 2015). In this respect, the explorative study conducted by Sestito et al. provide support for a complex interaction between anomalous self-experiences and psychotic symptoms in the context of neutral stimuli misperception in schizophrenia. These preliminary findings outline some testable perspectives on the connection between molecular neurochemistry of delusions formation at a brain level and their psychopathological corollary. Gallager and Trigg illustrate the relevance of phenomenological accounts of schizophrenia and agoraphobia, highlighting the importance of considering the interdependent nature of neural aspects, subjective experience, and social environment. In the work of Haug et al., results describe how anomalous self-experiences might be a useful target in other psychopathological conditions like depression, to assist the clinician in understanding patients' experience of self-esteem to prevent suicidality. Taken together, these studies show the potential of applying anomalous self-experiences as a target phenotype for neurobiological and genetic research in the context of schizophrenia and other psychopathological diseases.

Further theoretical efforts directed at exploring the connections between anomalous self-experiences and the brain substrate are presented in the works of Kuang and Jalal and Ramachandran. The paper of Kuang proposes a unified social motor cognition theory in order to conceal the neural and the mental levels of cognitive processing in the context of the mirror-touch synaesthesia manifestations. The neural level is herein considered the physical process regarding basic sensory-motor aspects of the action, which supports motor imitation and goal understanding (i.e., the Mirror Neuron System, MNS) whereas, the mental level concerns the attribution of mental states, which supports inferring others' minds and self-other distinctions. In the work of Jalal and Ramachandran for example, the MNS substrate is suggested to play a role in giving rise to a particular sort of out of body experiences occurring during the REM sleep. Such experiences include sensing and seeing the presence of threatening intruders in one's bedroom—the so called “ghostly bedroom intruder” experience. According to these authors, in this condition the activation of the MNS would enable to see the world from an allocentric perspective, without leaving one's own body.

Further efforts are needed to indentify comprehensive protocols capitalizing upon the integration between the phenomenological and the (neuro) physiological levels of analysis. In this respect, the embodied cognition approach—considering the MNS as a neural substrate—offers an insightful perspective to inspire future research protocols aimed at bridging the body-object and the body-subject. To pursue this endeavor, it will be critical to unravel the (neuro) physiological mechanisms enabling the integration between inner body signals and exteroceptive inputs. The topic “Embodying the self: neurophysiological perspectives on the psychopathology of anomalous bodily experiences” is a very active research topic that has a major importance in providing advances for intervention approaches and for the understanding of vulnerability markers to enhance early identification of psychopathological diseases.

## Author contributions

MS: Intellectual conceptualization, literature review, and manuscript drafting. AR: Intellectual conceptualization and literature review. GS: Literature review. VG: Intellectual conceptualization, literature review, and manuscript drafting supervision. All the authors contributed to the final revision of the manuscript.

### Conflict of interest statement

The authors declare that the research was conducted in the absence of any commercial or financial relationships that could be construed as a potential conflict of interest.
